# Synthetic MRI in breast cancer: differentiating benign from malignant lesions and predicting immunohistochemical expression status

**DOI:** 10.1038/s41598-023-45079-2

**Published:** 2023-10-20

**Authors:** Xiaojun Li, Zhichang Fan, Hongnan Jiang, Jinliang Niu, Wenjin Bian, Chen Wang, Ying Wang, Runmei Zhang, Hui Zhang

**Affiliations:** 1 Department of Medical Imaging, Shanxi Medical University, Taiyuan, Shanxi China; 2 Department of Radiology, Cancer Hospital Chinese Academy of Medical Sciences, Shenzhen Center, Shenzhen, China; 3 Department of Breast Surgery, Cancer Hospital Chinese Academy of Medical Sciences, Shenzhen Center, Shenzhen, China; 4 Department of Radiology, The 2nd Affiliated Hospital of Shanxi Medical University, Taiyuan, Shanxi China; 5 Department of Pathology, The 2nd Affiliated Hospital of Shanxi Medical University, Taiyuan, Shanxi China; 6 Department of Radiology, First Hospital of Shanxi Medical University, No. 85, South Jiefang Road, Yingze District, Taiyuan, 030001 Shanxi China

**Keywords:** Breast cancer, Cancer, Cancer imaging

## Abstract

To evaluate and compare the performance of synthetic magnetic resonance imaging (SyMRI) in classifying benign and malignant breast lesions and predicting the expression status of immunohistochemistry (IHC) markers. We retrospectively analysed 121 patients with breast lesions who underwent dynamic contrast-enhanced magnetic resonance imaging (DCE-MRI) and SyMRI before surgery in our hospital. DCE-MRI was used to assess the lesions, and then regions of interest (ROIs) were outlined on SyMRI (before and after enhancement), and apparent diffusion coefficient (ADC) maps to obtain quantitative values. After being grouped according to benign and malignant status, the malignant lesions were divided into high and low expression groups according to the expression status of IHC markers. Logistic regression was used to analyse the differences in independent variables between groups. The performance of the modalities in classification and prediction was evaluated by receiver operating characteristic (ROC) curves. In total, 57 of 121 lesions were benign, the other 64 were malignant, and 56 malignant lesions performed immunohistochemical staining. Quantitative values from proton density-weighted imaging prior to an injection of the contrast agent (PD-Pre) and T2-weighted imaging (T2WI) after the injection (T2-Gd), as well as its standard deviation (SD of T2-Gd), were valuable SyMRI parameters for the classification of benign and malignant breast lesions, but the performance of SyMRI (area under the curve, AUC = 0.716) was not as good as that of ADC values (AUC = 0.853). However, ADC values could not predict the expression status of breast cancer markers, for which SyMRI had excellent performance. The AUCs of androgen receptor (AR), estrogen receptor (ER), progesterone receptor (PR), human epidermal growth factor receptor 2 (HER-2), p53 and Ki-67 were 0.687, 0.890, 0.852, 0.746, 0.813 and 0.774, respectively. SyMRI had certain value in distinguishing between benign and malignant breast lesions, and ADC values were still the ideal method. However, to predict the expression status of IHC markers, SyMRI had an incomparable value compared with ADC values.

## Introduction

Breast cancer, the most common malignancy in women, overtook lung cancer globally for the first time in 2020^1^. Immunohistochemistry (IHC) can reflect the internal heterogeneity of tumour, which is an essential basis for determining tumour subtypes, judging prognosis and guiding treatment plans^2,3^. The expression of sex hormone receptors determines whether breast cancer patients need to receive internal treatment. In recent years, the role of androgen receptors (AR) in breast cancer has gradually attracted attention^4^. P53, Ki-67 and human epidermal growth factor receptor 2 (HER-2) are closely related to the proliferation state of the cells and are important indicators for monitoring therapeutic effect^5,6^.

Correctly evaluating benign and malignant lesions and immunohistochemistry results is highly important for patients. At present, the clinical imaging methods commonly used to evaluate breast lesions include ultrasound, mammography, and magnetic resonance imaging (MRI)^7–9^. Among them, MRI has become the primary method because of its excellent spatial and soft-tissue resolution. In addition, dynamic contrast-enhanced MRI (DCE-MRI) can intuitively reflect the real-time blood supply of neoplasms with good time resolution and plays a vital role in the grading of breast lesions^10–12^. With the recent controversy and the concerns about the renal fibrosis of gadolinium containing contrast agents, the recommendations are that “gadolinium based contrast agents should only be administered if the information so provided is necessary, and specifically expected to increase the confidence in correct disease diagnosis or assessment thereof, or disease exclusion^10–12^.

Synthetic MRI (SyMRI) is a new technology for quantifying the values of T1-, T2- and proton density (PD)-weighted imaging (PDWI). Using only a multi-dynamic multi-echo (MDME) sequence in one scan, T1WI, T2WI, PDWI, and inversion recovery imaging can be performed, and T1, T2, and PD maps can be obtained at the same time, which significantly reduces the scanning time to increase clinical utility^13,14^. Relevant studies have shown that T2 values acquired from SyMRI are reliable and are not significantly different from those acquired with traditional multi-echo spin-echo (MESE) sequence technology^15^. SyMRI technology has been suggested to have clinical value in many fields, such as to image the nervous and musculoskeletal systems^14,16–21^.

Diffusion-weighted imaging (DWI) is a critical sequence that can obtain lesion characteristics without media as SyMRI. The apparent diffusion coefficient (ADC) is one of the parameters of DWI. The ADC value measures the Brownian motion of water molecules in the tissue^22^. Many previous studies have demonstrated the difference in ADC values between benign and malignant lesions in the breast. Studies have shown that for BI-RADS category 4 lesions with overlapping benign and malignant neoplasms, ADC values can be used for risk stratification to downgrade BI-RADS category 4 lesions to reduce the number of unnecessary invasive examinations^23–25^. However, the low resolution of ADC maps has always been the biggest obstacle to its clinical use.

Therefore, the purpose of this study was to evaluate and compare the diagnostic performance of parameters of SyMRI and DWI in classifying benign and malignant breast lesions. And predicting the expression status of IHC markers among malignant lesions with that of ADC values.

## Materials and methods

### Patient selection

**Figure 1:**
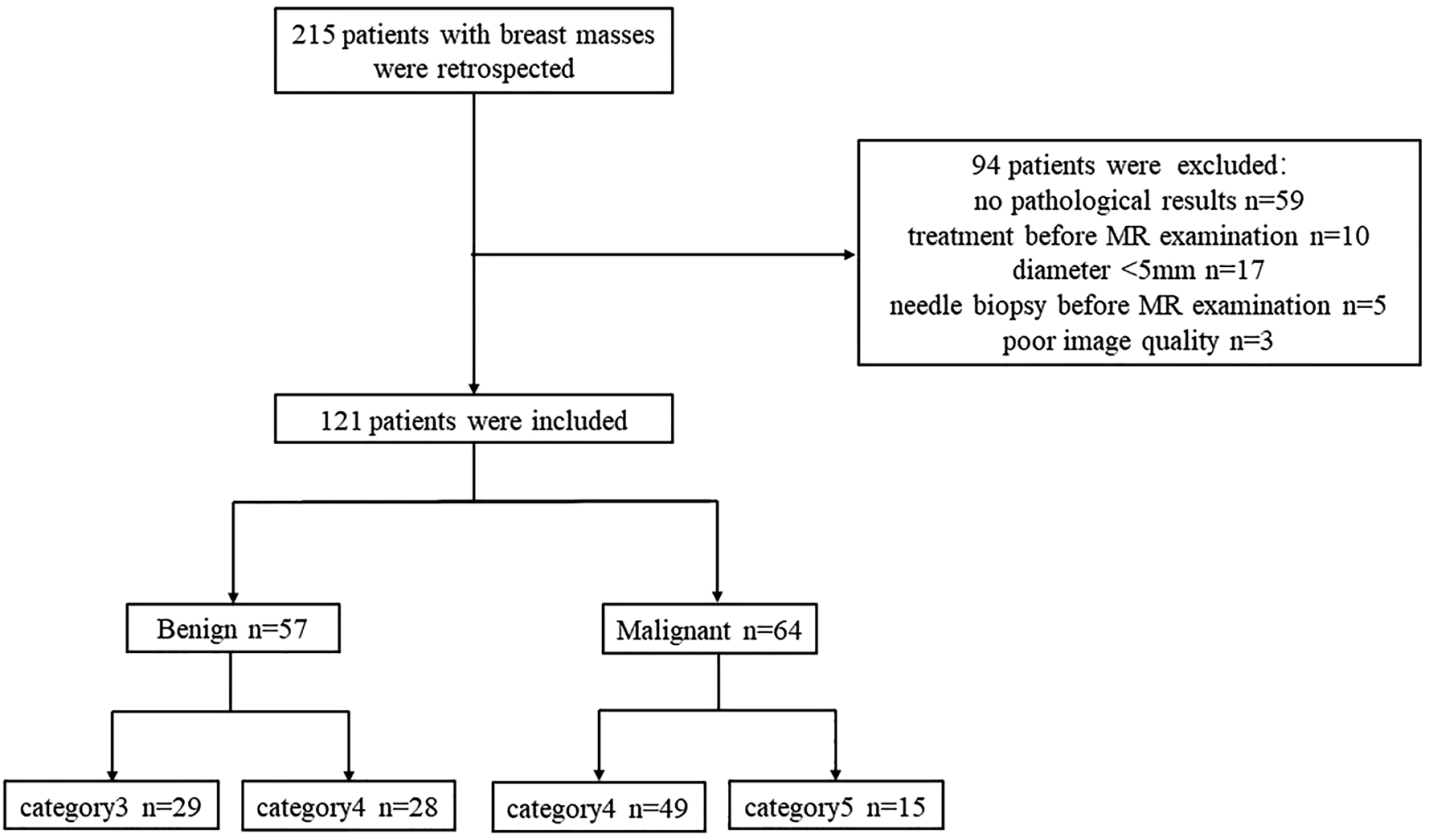
Flowchart showing the patient selection process to form the study sample.

This was a retrospective study that was approved by the ethics review committee of Second Hospital of Shanxi Medical University, and the requirement for informed consent was waived. All methods were performed in accordance with the relevant guidelines and regulations. The study flowchart is shown in Fig. 1. We analyzed patients with breast lesions who underwent MRI examinations before surgery in our hospital from January 2019 to December 2020. The inclusion criteria were as follows: underwent 3.0 T MRI before surgery, including SyMRI, DCE-MRI, and DWI; had images of good quality without obvious artefacts; did not receive chemotherapy or radiotherapy before MRI; did not undergo needle biopsy before MRI, and had a clear pathological diagnosis. The exclusion criteria were as follows: did not undergo pathological examinations in our hospital; had lesions with diameters less than 5 mm, making it difficult to delineate the region of interest (ROI); and had a lesion undetectable with the magnetic resonance imaging compilation (MAGiC) sequence.

### MRI acquisition

All patients underwent breast examinations performed with a 3.0 T MRI scanner (Signa Pioneer, GE Healthcare, WI, USA). Patients were examined in the prone position using an 8-channel phased-array breast surface coil. The examination included a conventional breast sequence and axial MAGiC sequence. The axial DWI (b_1_ = 0, b_2_ = 800) used a single-shot echo-planar imaging (SS-EPI) technology with the repetition time (TR) = 4137 ms; echo time (TE) = 61.7 ms; field of view (FOV) = 32 cm, matrix = 128 × 128. Axial DCE-MRI used a 3D dual-echo spoiled gradient recalled echo (SPGR) T1-weighted sequence called Differential Subsampling with Cartesian Ordering (DISCO) by GE company and fat suppression using DIXON technology. The MAGiC sequence is based on MDME sequence, which selected a cross-layer selection saturation pulse and multiple echo acquisition. The parameters were as follows: scan time = 4 min and 37 s; TR = 4617 ms; TE = 10.2/91.9 ms; the total number of slices = 23, slice thickness = 5.0 mm, slice interval = 1.0 mm, FOV = 32 cm, and matrix = 320 × 256. The scanning order was T1WI, T2WI, DWI, first MAGiC sequence, DCE and second MAGiC sequence. The second MAGiC sequence was acquired after DCE-MRI, 9 min and 52 s after the injection of the contrast agent.

### MRI analysis

The radiologist A and B with 20 years of experience were selected to detect lesions independently on DCE-MRI, without any knowledge of pathological results. Then, the ROI (a box with size of 0.5 cm × 0.5 cm) was outlined on the MAGiC sequences (before and after enhancement) and ADC maps in the layer with the maximum major axis. When delineating the ROI, the solid part of the mass was outlined to avoid bleeding and cystic areas. The postprocessing software automatically calculated the T1, T2, PD and ADC values of all voxels in the ROI. Among them, the standard deviation (SD) represented the degree of dispersion of all voxel-related quantitative values in the ROI, and Pre and Gd represented the quantitative values before and after enhancement, respectively. Finally, T1-Pre, T2-Pre PD-Pre, SD of T1-Pre, SD of T2-Pre, SD of PD-Pre, T1-Gd, T2-Gd, PD-Gd, SD of T1-Gd, SD of T2-Gd, and SD of PD-Gd values were obtained from MAGiC sequence. ADC maps were automatically converted from DWI scans; ADC values were calculated with the formula ADC = ln(SI0/SI1)/(b1 − b0), where SI represents the intensity of the tissue signal on DWI with the corresponding b value. When the lesions outlined by the two radiologists were in agreement, the average of all of the quantitative values was calculated. If the lesions were inconsistent, the radiologist C (director) judged and re-outlined the lesions.

### Pathologic assessment

All pathological results came from our hospital’s electronic case system, including microscopic findings and immunohistochemical results. Pathological sections were stained with haematoxylin and eosin. IHC was performed using streptavidin-peroxidase to detect the expression levels of AR, estrogen receptor (ER), progesterone receptor (PR), HER-2, P53, and Ki-67 in malignant lesions. Each IHC index was divided into high expression and low expression groups according to the actual expression level; AR ≥ 80%, ER ≥ 80%, PR ≥ 70%, Ki-67 > 14%, and HER-2 graded as 2 + or high was defined as high expression, otherwise, the tissues were stratified into the low expression group^20,26,27^. The grouping criteria of AR, ER and PR based on on distribution of the data.For P53, the presence of a mutation and diffuse strong positive expression was defined as high expression, while wild-type P53 and partially positive expression were defined as a low expression^28^. All the pathological data above were from surgical specimens.

### Statistical analysis

Data were analysed using SPSS (Version 26.0, IBM, USA), and P < 0.05 was considered to indicate a significant difference. Receiver operating characteristic (ROC) curves were drawn on GraphPad Prism (Version 8.0, GraphPad Software, USA). The Shapiro–Wilk test was used to test if the data followed a normal distribution. Normally distributed data are represented as the mean and standard deviation, and nonnormally distributed data are defined as the median. The mann–Whitney U test or t test was used to compare the difference between two groups for continuous data, while Chi-square test was used to compare the discerte data.

Multivariate analysis was performed using logistic regression, and the backward LR method was used to filter the variable values. ROC curves were used to test the ability of SyMRI and ADC values to classify breast lesions and predict the expression status of IHC markers. The area under the curve (AUC), 95% confidence interval (CI), sensitivity, and specificity were the evaluation indicators. The Z test was used to identify significant differences in AUC.

## Results

We collected a total of 215 patients with breast masses between January 2019 and December 2020. All patients were female. The results are shown in Fig. 1. Finally, 94 patients were excluded, including 59 patients with no pathological results, 10 with a history of treatment, 17 patients with lesions with a diameter < 5 mm, 5 who underwent needle biopsy before the examination, and 3 with poor image quality. A total of 121 patients were retained, aged 16–70 years, and classified as BI-RADS category 3–5 after MRI. No benign lesions were rated as category 5, and no malignant lesions were rated as category 3. Fifty-seven lesions were benign (29 category 3, 28 category 4), including 42 cases of breast fibroadenomas (glandular hyperplasia with fibroadenomas, simple fibroadenomas), 9 cases of intraductal papilloma, 5 cases of inflammatory granuloma, and 1 case of phyllodes neoplasm. There were 64 breast cancers (15 category 5, 49 category 4), most of them were non-specific invasive cancer (56 lesions), with 3 being a specific carcinoma (2 papillary carcinomas and 1 medullary carcinomas), and 3 cases were non-invasive cancer, which was all ductal carcinoma in situ. Two were rare tumours: spindle cell carcinoma and high-grade sarcoma. In the malignant group, 56 lesions had successful immunohistochemical staining.

**Figure 2:**
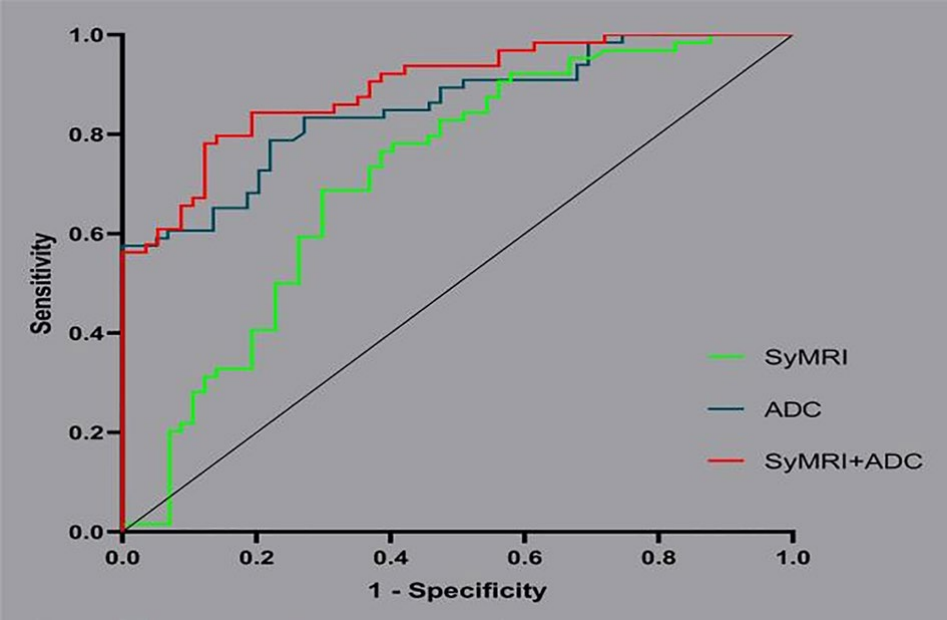
ROC curves based on SyMRI and ADC values for differentiating between benign and malignant breast lesions.

**Figure 3:**
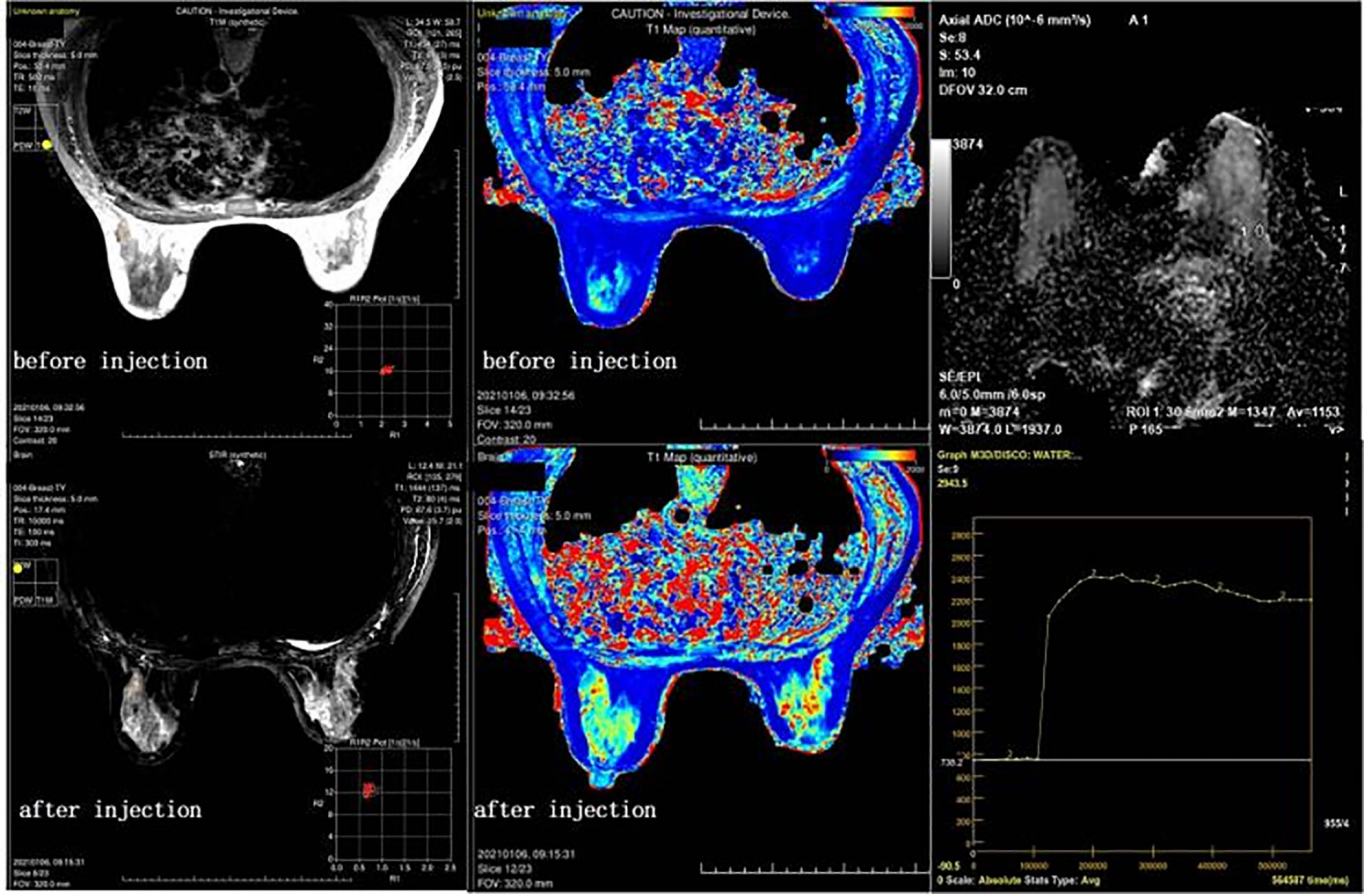
Images before and after enhancement of a patient who was confirmed to have intraductal papilloma. The DCE curve showed a plateau, and the average ADC value was 1.153 × 10^−3^ mm^2^/s. The lesion was classified as BI-RADS 4 preoperatively. The lesion’s SD of PD-Pre, T2-Gd, and SD of T1-Gd were 4.5 ms, 80 ms, and 1444 ms, respectively, and the lesion was diagnosed as benign by the model combining SyMRI and ADC values.

**Figure 4:**
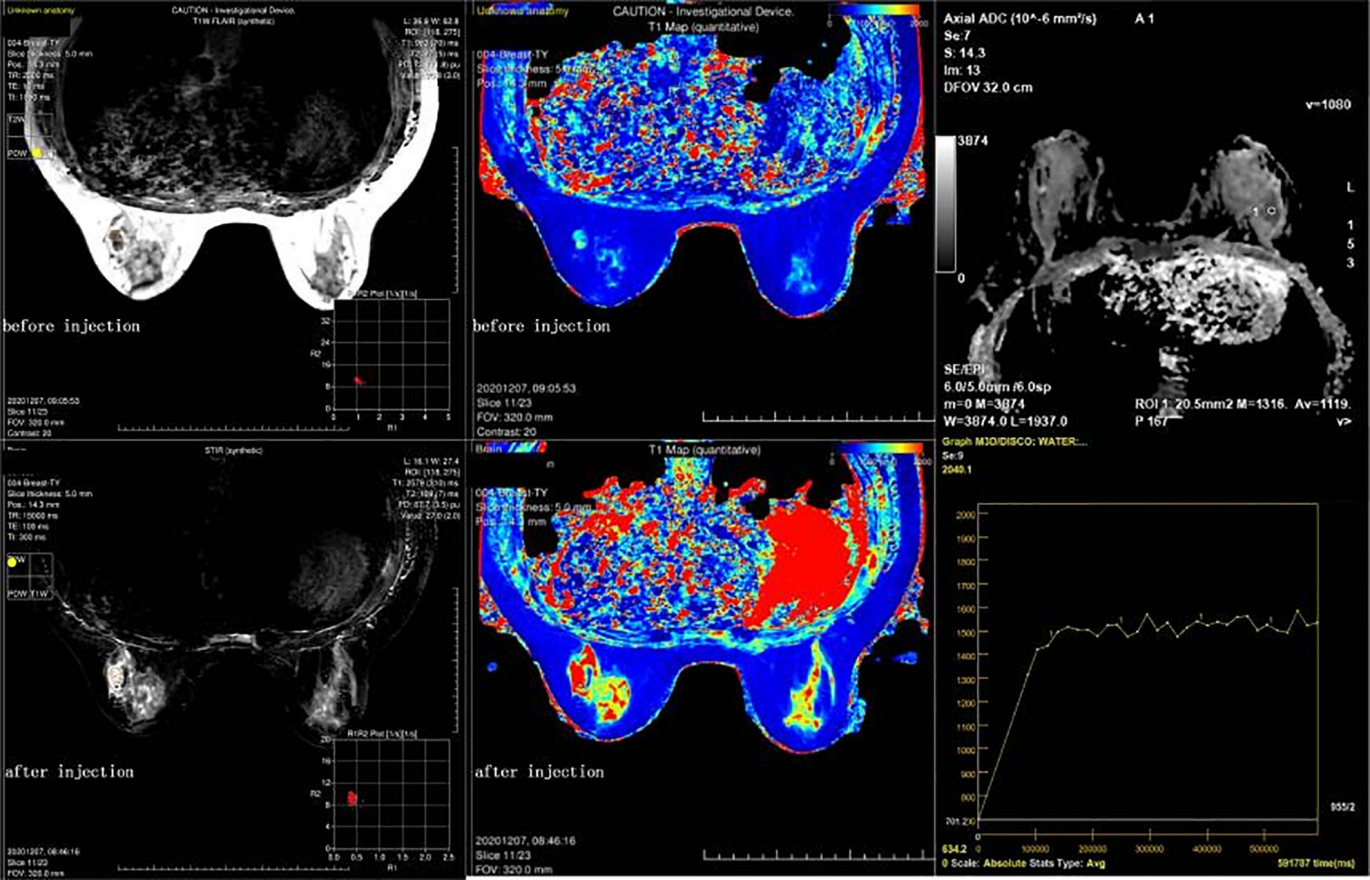
Images before and after enhancement of a patient who was confirmed to have invasive ductal carcinoma. The DCE curve showed a plateau, and the average ADC value was 1.119 × 10^−3^ mm^2^/s; the lesion was classified as BI-RADS 4 preoperatively. The lesion’s SD of PD-Pre, T2-Gd, and SD of T1-Gd were 1.8 ms, 109 ms, and 2579 ms, respectively, and the lesion was diagnosed as malignant by the model combining SyMRI and ADC.

The T1-Gd, SD of T1-Gd, SD of T1-Pre, and SD of T2-Pre did not follow a normal distribution. The other variables obeyed a normal distribution. The SD of PD-Pre, T2-Gd, and SD of T1-Gd were the SyMRI parameters helpful in distinguishing between benign and malignant breast lesions (Table 1). The mean ADC value of benign lesions was 1.38 × 10^–3^ mm^2^/s, and that of malignant lesions was 0.97 × 10^–3^ mm^2^/s. There were no benign lesions with an ADC value less than 1 × 10^–3^ mm^2^/s. However, the classification ability of SyMRI was not as good as that of ADC values (AUC = 0.716 VS AUC = 0.853, *p* = 0.018, Table 2). The combination of SyMRI with ADC value had the best classification ability but was not significantly different from ADC (AUC = 0.895 VS AUC = 0.853, *p* = 0.337, Table 2). Moreover, the sensitivity and specificity of the combined methods were also the highest, at 0.781 and 0.877, respectively. We used the probability of the best cut-off point on the ROC curve of SyMRI combined with ADC as the critical value to classify the lesions with BI-RADS scores of 4: 79% of benign lesions (22/28) and 61% of malignant lesions (30/49) were accurately screened. The ROC curves are shown in Fig. 2. Figure 3 and Fig. 4 showed the application of SyMRI parameters and ADC value in benign and malignant lesions.

**Table 1:** The comparison of SyMRI parameters and ADC value in benign and malignant lesions.

	Benign	Malignant	*P*	95%CI
SD of PD-Pre	4.72 ± 2.55	4.01 ± 1.98	0.045	0.598–0.994
T2-Gd	82 ± 24	73 ± 13	0.002	0.925–0.983
SD of T1-Gd	41	58	0.049	1.000–1.015
ADC	1.38 ± 0.26	0.97 ± 0.29	< 0.001	0.000–0.022

The normally distributed data are represented as the mean ± standard deviation.

The nonormally distributed data are represented as the media.

*P*-value < 0.05 is statistically significant.

Pre: before injection; Gd: after injection; SD: standard deviation for quantitative values within ROI.

**Table 2:** The performance of SyMRI parameters and ADC value in distinguishing benign from malignant breast lesions.

	AUC	95%CI	*P*-Value	Sensitivity	Specificity	Z-test
ADC	0.853	0.789–0.918	< 0.001	0.576	1.000	*P*(ADC-SyMRI) = 0.018
SyMRI	0.716	0.622–0.810	< 0.001	0.688	0.702	*P*(ADC-SyMRI + ADC) = 0.337
SyMRI + ADC	0.895	0.840–0.949	< 0.001	0.781	0.877	*P*(SyMRI-SyMRI + ADC) = 0.001

*P*-value < 0.05 is predictive.

**Figure 5:**
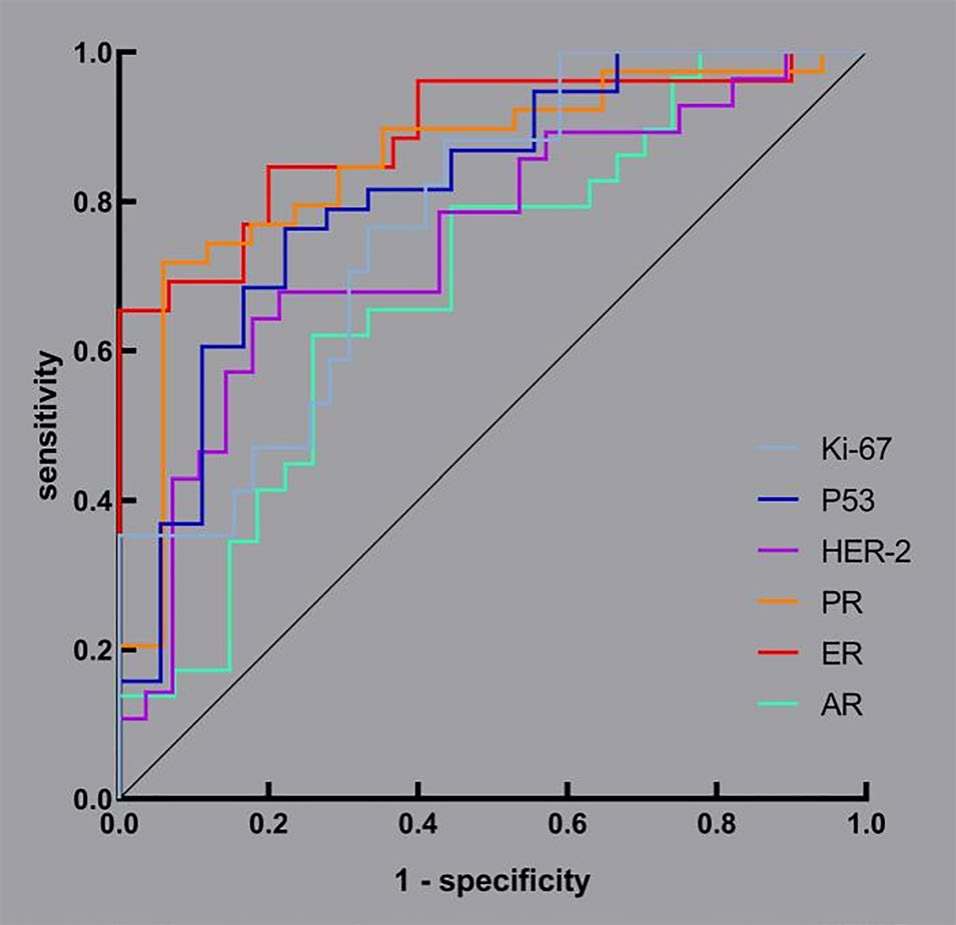
ROC curves based on SyMRI for predicting the expression of IHC markers including AR, ER, PR, Her-2, P53, and Ki-67.

Table 3 shows the SyMRI parameters related to IHC expression status (56 cases of nonspecific invasive cancer). The ADC value was not significantly different between the high and low expression groups for all 6 IHC indicators. Table 4 shows the effectiveness of SyMRI in predicting the expression of the IHC indicators. Logistic regression analysis showed that T2-Pre, T2-Gd, SD of T2-Gd, and SD of PD-Gd were closely related to the expression status of ER, with an AUC that could reach 0.890, 95% CI of 0.801–0.979, the sensitivity of 65.4% and specificity of 100%. The ability to predict AR was the weakest among the six indicators, with an AUC of only 0.687, 95% CI of 0.547–0.827, the sensitivity of 62.1%, and specificity of 74.1%; only PD-Gd was significantly different between groups. The ROC curves are shown in Fig. 5.

**Table 3:** SyMRI compared in IHC low and high expression of breast cancers.

	SyMRI	Low expression	High expression	*P*	95%CI
AR	PD-Gd	86.59 ± 18.92 (n = 29)	77.64 ± 16.36 (n = 27)	0.042	0.922–0.999
ER	T2-Pre	95.54 ± 16.50 (n = 25)	88.48 ± 35.52 (n = 31)	0.045	1.001–1.069
	T2-Gd	81.18 ± 12.88	67.79 ± 10.91	0.002	0.759–0.941
	SD of T2 Gd	5.94 ± 2.87	5.24 ± 2.69	0.049	0.402–0.998
	SD of PD Gd	4.05 ± 2.63	5.90 ± 3.18	0.011	1.131–2.631
PR	T2-Gd	76.45 ± 13.15 (n = 39)	67.62 ± 12.62 (n = 17)	0.011	1.027–1.229
	SD of T2 Pre	6.00	4.50	0.004	0.733–0.941
HER-2	PD Pre	71.62 ± 17.82 (n = 28)	78.30 ± 11.55 (n = 28)	0.010	1.021–13,161
	SD of T1 Pre	147.75	153.50	0.040	0.990–1.000
P53	T2 Pre	92.68 ± 33.27 (n = 38)	89.42 ± 15.33 (n = 18)	0.048	0.794–0.999
	SD of T2 Gd	5.28 ± 2.56	6.14 ± 3.16	0.036	1.029–2.351
	SD of PD Gd	5.52 ± 3.31	4.13 ± 2.25	0.021	0.345–0.917
Ki67	T1 Pre	1632.00 ± 666.95 (n = 18)	1526.31 ± 351.15 (n = 38)	0.028	1.000–1.006
	SD of T1 Pre	206.50	147.00	0.011	0.976–0.997
	PD Gd	90.21 ± 22.57	78.81 ± 14.88	0.028	0.927–0.996

*P*-value < 0.05 is statistically significant.

Pre: before injection; Gd: after injection; SD: standard deviation for quantitative values within ROI; AR: androgen receptor; ER: estrogen receptor; PR: progesterone receptor; HER-2: human epidermal growth factor receptor 2; P53: P53 protein; Ki-67: Ki-67 protein.

**Table 4:** SyMRI’s ability to predict the expression of IHC.

	AUC	95%CI	Sensitivity	Specificity	*P*-Value
AR	0.687	0.547–0.827	0.621	0.741	0.016
ER	0.890	0.801–0.979	0.654	1.000	< 0.001
PR	0.852	0.741–0.963	0.718	0.941	< 0.001
HER-2	0.746	0.616–0.877	0.643	0.821	0.002
P53	0.813	0.689–0.937	0.763	0.778	0.002
Ki-67	0.774	0.648–0.899	0.882	0.564	0.001

*P*-value < 0.05 is predictive.

AR: androgen receptor; ER: estrogen receptor; PR: progesterone receptor; HER-2: human epidermal growth factor receptor 2; P53: P53 protein; Ki-67: Ki-67 protein.

## Discussion

In this study, we comprehensively evaluated the value of SyMRI in the diagnosis and treatment of breast lesions, including the classification of benign and malignant lesions and the prediction of the expression status of IHC markers. Furthermore, the parameters of SyMRI were compared with ADC values. We found that paremeters of SyMRI were limited in the classification of benign and malignant lesions but had an excellent performance in analyzing the differences in expression of IHC status of lesions. In contrast, although ADC values were very helpful for the classification of lesions, there was no significant effect on in predicting the expression of different receptors in our experiment.

AR, ER, and PR are all expressed in normal breast tissues. In tumour cells, the degree of deletion is a response to atypia, and their expression levels are also an important basis for endocrine therapy^2,4,29,30^. Her-2, P53, and Ki-67 are important indicators of tumour proliferation status and are very important for predicting prognosis^6,31–33^. However, there is no clear noninvasive method to evaluate the above indicators. Therefore, we tried to use SyMRI and ADC values to evaluate the expression status of IHC markers and achieved significant results, but there were also discrepancies with previous studies. First, regarding Ki-67, the AUC value was lower than that of Matsuda et al.^20^. We speculate that the difference may be due to the imbalance of the two groups of people; therefore, the results need further confirmation. In their experiment, the SDs of T1-Gd and T2-Gd were significantly different between groups, but we applied T1-Pre and the SDs of T1-Pre and PD-Gd. Some reports have shown that the T1 value is indeed a parameter highly related to Ki-67^33,34^. Second, we found that T2 and its related quantitative values were more likely to predict the expression of IHC indicators, especially ER, and 3 of the 4 parameters were related to T2. Moreover, SyMRI had the best performance in predicting ER expression status, with an AUC reaching 0.890. However, Mirinae Seo’s experimental results showed that T2* was not associated with the expression level of sex hormone receptors^35^. Although T2 and T2* are two interrelated indicators, T2* reflects the influence of inhomogeneous magnetic fields, so the results are still up for discussion. Similarly, the results regarding ADC values need further confirmation. SY Choi^36^ used univariate analysis to prove that the ADC value is correlated with the expression level of IHC markers^37^. However, in our experiment, the ADC values were not significantly different among the six IHC indicators. We believe that compared to multivariate analysis, the univariate analysis may not be as reliable. Matsuda’s^20^ experiments also proved this point.

In the classification of benign and malignant breast lesions, the AUC (0.7161) of SyMRI was similar to the results of Megumi Matsuda et al.^19^. The performance of the ADC value was predictably stable, and there was no obvious difference from previous research results^38^. The diagnostic power of SyMRI was not as good as that of the ADC value (AUC = 0.8532). However, the tissue contrast and spatial resolution of ADC maps were low, leading to certain limitations in clinical applications. Additionally, we found that the logistic regression model of ADC combined with SyMRI showed excellent diagnostic performance. The AUC reached 0.8945, which is higher than the model combining SyMRI and DCE-MRI from Megumi Matsuda (AUC = 0.831)^19^. As shown in Figs. 4 and 5, the ADC values of the benign and malignant lesions were similar, and the DCE curves also showed plateaus. It was difficult to distinguish between benign and malignant lesions when relying on traditional imaging data. However, the model using SyMRI combined with ADC could accurately predict the nature of the lesion. There were also some differences between our results and those in other studies; SD of PD-Pre, T2-Gd, and SD of T1-Gd were statistically significant predictors in our study, in contrast to T1-Pre and T2-Pre in Tiebao Meng’s experiment^14^ or T1-Pre only in Megumi Matsuda’s study^19^. After analysis, we believe that there are two reasons for these differences: first, the composition of the breast is complex, and fat, fibre, and glands will affect T1-Pre and T2-Pre^39^; and second, the fluctuation of hormones altered the signal of the breast. Compared with that in developed countries, the highest incidence of breast cancer in Chinese women is found in a younger population, between 45 and 55 years old; most of these women are perimenopausal, and their hormone levels fluctuate greatly. This may be another reason for the differences in T1-pre and T2-Pre^40^.

PDWI mainly reflects the difference in proton content between different tissues per unit volume. In human tissues, the generation of MR signals in nonfat tissues mainly depends on the hydrogen protons in water molecules. Therefore, the PD value actually mainly reflects the water molecule content in the tissue. However, unlike the T2 value, the PD value reflects the total amount of free water and bound water. In our experiment, the SD of the PD-Pre value of malignant lesions was smaller than that of benign lesions, but the PD value itself was not different between the two groups, indicating that the distribution of water molecules in malignant neoplasms was more concentrated. In other words, the total amount of water molecules in benign and malignant neoplasms was equivalent, but malignant neoplasm cells were rich in the cytoplasm and had high cell density, so the bound water content was high, while the free water content was low^15,38^. To the best of our knowledge, this is the first time that the SD of PD-Pre has been found to have value in differentiating between benign and malignant lesions in the breast. In the existing literature, the PD value is reported to be mainly related to the skeletal system. Yuki Arita^41^ found that among lesions of bone metastases of prostate cancer, the PD value of active lesions was greater than that of inactive lesions. We speculate that in bones, in addition to water, the hydrogen protons from fat in the marrow contribute to MR signals. Therefore, when the disease occurs in bone, the PD value changes significantly.

T2-GD and SD of T1-GD were quantitative values that were significantly different after enhancement in previous studies. This seems to be inconsistent with our impression because we observed the enhancement effect of the contrast agent on the T1WI most of the time. Therefore, there should be a significant difference in the T1-GD value, in theory. After analysis, we believe that this observation may be related to the scan time after injection and the concentration of the local contrast agent. Akifumi Hagiwara’s research shows that the acquisition time of SyMRI after drug injection will affect the degree of enhancement of the lesion on T1WI. Among the possible scan times, scanning at 1 min and 30 min after injection achieved better contrast, while the contrast of scans acquired at approximately 10 min was poor^42^. Gadodiamide is a paramagnetic extracellular contrast agent that can shorten the tissue T1 and T2 relaxations. When the concentration of the contrast agent is low, the original longitudinal relaxation time of body tissue is longer, so the T1 value changes more significantly than the T2 value. As the concentration increases, the T2 value shortens gradually, and eventually, its shortening will make it lower than the T1 value, which is the so-called negative enhancement of contrast agents^42,43^. Bothe we know, when the paramagnetic contrast agent reaches a high concentration, the change of transverse relaxation will be more obvious than that of longitudinal relaxation. In this experiment, the enhanced MAGiC sequence started after the dynamic enhancement sequence, 9 min and 52 s after the contrast agent injection. At this time, the concentration of the contrast agent in the lesion has accumulated to a relatively high concentration, so the T2-GD value was more prominent than the T1-GD value. The scan time used by Megumi Matsuda^19^ was 12 min after the injection, which was later than that for our scans. Perhaps this is one reason why our experimental results are different. In addition, the SD of T1-GD of malignant lesions was larger than that of benign lesions, which may be caused by the more scattered distribution of contrast agent in the tissue due to the abundant capillaries of malignant lesions.

There are still some limitations in our research. First, this was a single-centre study, and the sample size was still limited. Moreover, the proportion of benign and malignant diseases was uneven, the benign neoplasms were mostly fibroadenomas, and the number of triple-negative breast cancers among the malignant neoplasms was limited, which caused selection bias to a certain extent. Especially when analysing IHC markers, the number of people in different index groups was quite different. Second, the slice thickness and spacing of the MAGiC sequence scans were too large. Although we reduced the delineation range of the ROI as much as possible, it was still inevitable that non-neoplasm tissues were included, and the enhanced MAGiC sequence was implemented too late after the drug injection. Third, existing research showed that the expression of a hormone receptor > 1% could be considered positive^27^, so the cut-off values for hormone receptors in our study have no theoretical basis. Fourth, DWI adopted single-shot echo-planar imaging (SS-EPI), which reduces the scanning time but lowers the resolution. This may be one of the reasons for the limited value of ADC maps in predicting the expression status of IHC markers in this study. Finally, the ROI on the MAGiC sequence and ADC map cannot completely match the pathological biopsy area, which is an unavoidable systematic error.

## Conclusion

In general, our experiments prove that SyMRI has great potential in predicting the expression status of IHC markers of breast cancer and is expected to become an effective texture analysis tool. Although it has limited value in distinguishing between benign and malignant breast lesions, SyMRI can provide both high contrast images and quantitative maps after one scan with a shortened scan time. The above advantages make SyMRI a powerful tool in clinical diagnosis, treatment evaluation, and prognosis evaluation unmatched by traditional scanning sequences. SyMRI has great potential in evaluating diseases, and further experiments are still needed to prove this hypothesis.

## Data Availability

The datasets used and/or analysed during the current study available from the corresponding author on reasonable request.
